# Supplementary material for: Modeling glioblastoma heterogeneity as a dynamic network of cell states
Source: Mol Syst Biol. 2021 Sep 16;17(9):e10105. doi: 10.15252/msb.202010105 (PMC8444284; doi:10.15252/msb.202010105)
Supplement: Supplementary file 5 — Source Data for Figure 3 [file MSB-17-e10105-s001.zip › Figure3A_sourcedata/GSEA_3065/nosigdb_state6.GseaPreranked.1622718443993/SUVA_G2M_PMID_31327527.html]

Details for gene set SUVA\_G2M\_PMID\_31327527[GSEA]

|  || Dataset | state6 |
| Phenotype | NoPhenotypeAvailable |
| Upregulated in class | na\_neg |
| GeneSet | SUVA\_G2M\_PMID\_31327527 |
| Enrichment Score (ES) | -0.64143306 |
| Normalized Enrichment Score (NES) | -2.2282803 |
| Nominal p-value | 0.0 |
| FDR q-value | 0.0 |
| FWER p-Value | 0.0 |
Table: GSEA Results Summary

  

Fig 1: Enrichment plot: SUVA\_G2M\_PMID\_31327527      
 Profile of the Running ES Score & Positions of GeneSet Members on the Rank Ordered List

  

| PROBE | GENE SYMBOL | GENE\_TITLE | RANK IN GENE LIST | RANK METRIC SCORE | RUNNING ES | CORE ENRICHMENT || 1 | TTK |  |  | 2262 | 0.040 | -0.2467 | No |
| 2 | ANP32E |  |  | 2570 | 0.032 | -0.2724 | No |
| 3 | MZT1 |  |  | 2946 | 0.023 | -0.3084 | No |
| 4 | TMPO |  |  | 2978 | 0.022 | -0.3053 | No |
| 5 | PLK1 |  |  | 3116 | 0.020 | -0.3152 | No |
| 6 | HMGB3 |  |  | 3377 | 0.014 | -0.3409 | No |
| 7 | PSRC1 |  |  | 3406 | 0.013 | -0.3403 | No |
| 8 | RACGAP1 |  |  | 3432 | 0.012 | -0.3395 | No |
| 9 | CDC25B |  |  | 3887 | 0.003 | -0.3905 | No |
| 10 | CDC20 |  |  | 3996 | 0.001 | -0.4027 | No |
| 11 | RANGAP1 |  |  | 4700 | -0.013 | -0.4792 | No |
| 12 | CCNB2 |  |  | 4736 | -0.013 | -0.4793 | No |
| 13 | BUB1 |  |  | 5377 | -0.026 | -0.5448 | No |
| 14 | CDCA8 |  |  | 5535 | -0.029 | -0.5541 | No |
| 15 | ECT2 |  |  | 5560 | -0.030 | -0.5481 | No |
| 16 | KNSTRN |  |  | 5875 | -0.036 | -0.5734 | No |
| 17 | H2AFZ |  |  | 6079 | -0.040 | -0.5848 | No |
| 18 | SPAG5 |  |  | 6146 | -0.041 | -0.5801 | No |
| 19 | FOXM1 |  |  | 6684 | -0.055 | -0.6251 | Yes |
| 20 | TROAP |  |  | 6707 | -0.056 | -0.6111 | Yes |
| 21 | BUB1B |  |  | 6717 | -0.056 | -0.5954 | Yes |
| 22 | MXD3 |  |  | 6808 | -0.059 | -0.5883 | Yes |
| 23 | GPSM2 |  |  | 6953 | -0.063 | -0.5860 | Yes |
| 24 | TACC3 |  |  | 7022 | -0.066 | -0.5743 | Yes |
| 25 | CKAP2L |  |  | 7065 | -0.067 | -0.5594 | Yes |
| 26 | REEP4 |  |  | 7127 | -0.069 | -0.5461 | Yes |
| 27 | KIF11 |  |  | 7619 | -0.087 | -0.5764 | Yes |
| 28 | CDCA3 |  |  | 7631 | -0.087 | -0.5519 | Yes |
| 29 | AURKA |  |  | 7700 | -0.091 | -0.5328 | Yes |
| 30 | CCNB1 |  |  | 7958 | -0.104 | -0.5313 | Yes |
| 31 | KIFC1 |  |  | 7968 | -0.105 | -0.5012 | Yes |
| 32 | KPNA2 |  |  | 8045 | -0.110 | -0.4774 | Yes |
| 33 | CCNA2 |  |  | 8227 | -0.124 | -0.4614 | Yes |
| 34 | KIF4A |  |  | 8300 | -0.132 | -0.4305 | Yes |
| 35 | KIF2C |  |  | 8328 | -0.135 | -0.3936 | Yes |
| 36 | CKAP2 |  |  | 8330 | -0.136 | -0.3535 | Yes |
| 37 | TUBA1C |  |  | 8365 | -0.142 | -0.3154 | Yes |
| 38 | NCAPD2 |  |  | 8367 | -0.142 | -0.2734 | Yes |
| 39 | ARHGAP11A |  |  | 8513 | -0.164 | -0.2414 | Yes |
| 40 | TUBB4B |  |  | 8554 | -0.173 | -0.1946 | Yes |
| 41 | ARL6IP1 |  |  | 8628 | -0.203 | -0.1430 | Yes |
| 42 | CENPF |  |  | 8703 | -0.240 | -0.0803 | Yes |
| 43 | SMC4 |  |  | 8764 | -0.307 | 0.0037 | Yes |
Table: GSEA details [plain text format]

  

Fig 2: SUVA\_G2M\_PMID\_31327527: Random ES distribution      
 Gene set null distribution of ES for **SUVA\_G2M\_PMID\_31327527**
